# Fluorescence- and magnetic-activated cell sorting strategies to separate spermatozoa involving plural contributors from biological mixtures for human identification

**DOI:** 10.1038/srep36515

**Published:** 2016-11-18

**Authors:** Yan Xu, Jianhui Xie, Ronghua Chen, Yu Cao, Yuan Ping, Qingwen Xu, Wei Hu, Dan Wu, Lihua Gu, Huaigu Zhou, Xin Chen, Ziqin Zhao, Jiang Zhong, Rui Li

**Affiliations:** 1 School of Life Sciences, Fudan University, 2005 Songhu Road, Shanghai 200438, China; 2 Shanghai Key Laboratory of Crime Science Evidence, Key Laboratory of Forensic Evidence and Science Technology, Ministry of Public Security, Institute of Forensic Science, Shanghai Public Security Bureau, Shanghai 200083, China; 3 Department of Forensic Medicine, Shanghai Medical College, Fudan University, 138 Yixueyuan Road, Shanghai 200032, China

## Abstract

No effective method has been developed to distinguish sperm cells originating from different men in multi-suspect sexual assault cases. Here we combined MACS and FACS to isolate single donor sperm cells from forensic mixture samples including female vaginal epithelial cells and sperm cells from multiple contributors. Sperms from vaginal swab were isolated by MACS using FITC-conjugated A kinase anchor protein 3 (AKAP3) antibody; target individual sperm cells involving two or three donors were separated by FACS using FITC-labeled blood group A/B antigen antibody. This procedure was further tested in two mock multi-suspect sexual assault samples and one practical casework sample. Our results showed that complete single donor STR profiles could be successfully obtained from sperm/epithelial cell and sperm mixtures from two contributors. For unbalanced sperm/epithelial cells and sperm cells mixtures, sensitivity results revealed that target cells could be detected at as low as 1:32 and 1:8 mixed ratios, respectively. Although highly relies on cell number and blood types or secretor status of the individuals, this procedure would still be useful tools for forensic DNA analysis of multi-suspect sexual assault cases by the combined use of FACS and MACS based on sperm-specific AKAP3 antigen and human blood type antigen.

Short tandem repeat (STR) based individual identification from mixed samples remains a challenge in forensic science, particularly when the mixture contains only one cell type or the donors with the same gender^1^. In order to identify the suspect, unambiguous genotype analysis of mixture stains which contain cells from different individuals requires successful separation of the offender’s cells from those of the victim or others^2^.

Multi-suspect sexual assault is a crime frequently encountered by forensic scientists. The most common form of evidence is vaginal swabs containing epithelial cells from the female victim and sperms from different offenders. However, no effective method has been developed to successfully separate the offender’s cells from those of the victim and different perpetrators including a partner’s sperm from consensual sexual activity in these highly mixed samples. Therefore, to facilitate DNA typing and identification, it is an urgent task for forensic scientists to improve cell-separation methods for obtaining single-donor cell populations from a mixed sample.

In recent years, the immune-magnetic bead-based system, namely MACS (Magnetic-activated cell sorting), has been widely used for cell separation^3^. Based on immune-magnetic beads coupled with antibodies against sperm-specific antigens, MACS is fast, easy and economic. With a series of sperm membrane antigens found, previous studies have demonstrated that this technique can be used to isolate sperm cells from mixtures with epithelial cells^4^,^5^. In our study, we attempted to apply this technique to the separation of sperm cells from cell mixtures using antibody against sperm-specific antigen AKAP3 (A kinase anchor protein 3). Since the sperm-specific AKAP3 is exclusively expressed in the testis and is only detected in round spermatids^6^,^7^, AKAP3 is thought to be involved in spermatogenesis. Previous data showed AKAP3 located primarily in the sperm head and flagella, which may function as a regulator of both motility- and head-associated functions activities such as capacitation and the acrosome reaction^8^.

Cell sorting by flow cytometry, on the other side, is a means to sort cells differing in various parameters. This method is based on the labeling of cells with fluorescently tagged antibodies so that positive, dyed cells can be isolated from negative in a flow cytometer^9^,^10^. There has been limited studies using FACS (fluorescent-activated cell sorting) to separate cells from forensic mixtures, including sperm cells and epithelial cells mixtures^9^, and non-compromised blood and saliva mixtures^11^. More recently, Dean and colleagues exploits the intrinsic immunological variation among individuals to physically separate single donor cells in uncompromised whole blood mixtures by means of HLA antibody probes coupled to FACS^12^. In this study, we, for the first time, tested the feasibility of applying this technique for the isolation of single donor cells from mixed sperm cells involving plural contributors based on their ABO blood types.

Therefore, in our study, we combined MACS and FACS to isolate single donor sperm cells from forensic mixture samples including female vaginal epithelial cells and sperm cells from multiple contributors. Sequential use of the two methods include the extraction of spermatic DNA extraction from the vaginal swab by MACS based on sperm specific AKAP3 antibody binding and the separation of single donor sperm cells from cell mixtures involving plural contributors by FACS using ABO blood type antigen antibody. Our data indicates that these methods may be an effective approach for generating single donor STR profiles from forensic mixtures.

## Results

### ABO and FUT2 genotyping of sperm cells

Expression of ABO antigen in semen depends on the secretion status. Secretors, who have ABO antigens in their body secretions such as saliva, semen and etc., have at least one functional Se allele, whereas non-secretors, who fail to express ABO antigens in their secretions, are homozygous for the nonfunctional se allele. Homozygosity for null alleles at this locus occurs in approximately 20% of most populations^13^. Secretor type α (1, 2) fucosyltransferase gene (FUT2) blood group locus is responsible for the synthesis of soluble ABH blood group antigens in human body fluids^14^,^15^. Sequence analysis of FUT2 cDNA has revealed null alleles with a single base substitution in the protein coding region of FUT2 (A385T, G428A, C571T, C658T, G849A) were responsible for the inactivation of Se enzyme (See Supplementary Fig. 1)^14^,^15^. Thus, for sperm cells, the genotype identification of the secretors or non-secretors (FUT2 gene) is a prerequisite for this work.

All six ABO genotypes (O/O, B/B, A/A, A/O, A/B and B/O) could be determined by analyzing various combinations^16^. Mutations of FUT2 were screened by sequencing (See Supplementary Fig. 1). Out of 80 sperm samples, 13 were non-secretors and 67 were secretors in which 15 were A blood type, 18 were B blood type, 24 were O blood type and 10 were AB blood type.

### Western blot expression analysis of AKAP3 expression on sperm cells

**Figure 1:**
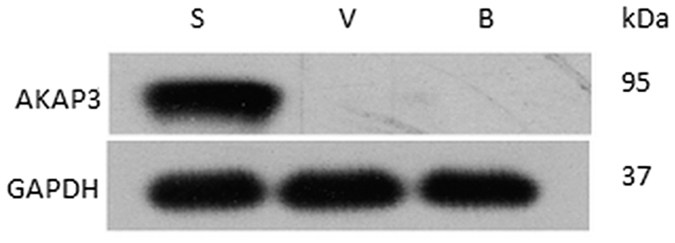
Western blot analysis of expression of AKAP3 in buccal and vaginal epithelial cells and sperm cells. Cropped gels/blots are displayed and full-length gels/blots are shown in the Supplementary Fig. 1. S: sperm cell, V: vaginal epithelial cell, B: buccal epithelial cell.

The AKAP3 protein, which has a molecular weight of 95 kDa, could be detected in sperm cells (Fig. 1). In contrast, the band was undetectable in buccal epithelial cells and vaginal epithelial cells.

### Immunofluorescent localization of AKAP3 on sperm cells

**Figure 2:**
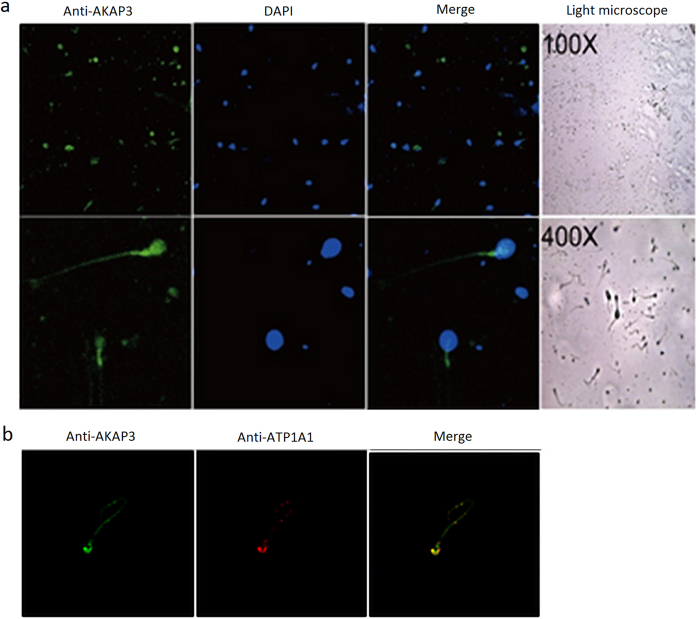
Immunofluorescent localization of AKAP3 in sperm cells. The expression and localization of AKAP3 in Sperm cells were detected with anti-AKAP3 monoclonal antibodies (**a**) and anti-ATP1A1 antibodies (**b**). Nuclei were counterstained with DAPI. Slides were observed under a laser scanning confocal microscope. Representative views were shown. Data were representative of 2 independent experiments.

To explore cellular localization of AKAP3, we stained sperm cells with immunofluorescent antibodies against AKAP3. Immunofluorescence analysis revealed that the signal of AKAP3 antibody was abundantly distributed on the membrane of sperm cells, mainly in the sperm head and tail (Fig. 2a); however, buccal and vaginal epithelial cells showed no immunofluorescence signal (data not shown). To further confirm this, we also double stained sperm cells with antibodies against AKAP3 and a cell membrane maker ATP1A1. ATP1A1, the catalytic component of NA+/K+-ATPase, is a ubiquitously expressed membrane protein and often used as the marker or internal control for plasma membrane protein. Double-immunostaining followed by fluorescence microscopy suggested a high degree of colocalization of AKAP3 and ATP1A1 in sperm cells (Fig. 2b).

**Figure 3:**
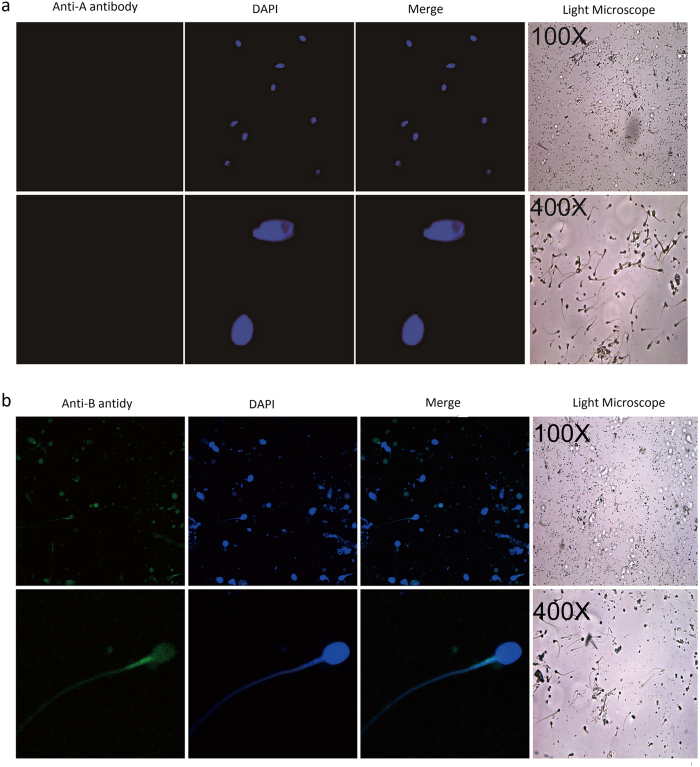
Immunofluorescent localization of blood group antigens in B-type sperm cells. The expression and localization of blood group antigen B in B-type sperm cells were detected with anti-blood group A antibody (**a**) and anti-blood group B antibody (**b**). Nuclei were counterstained with DAPI. Slides were observed under a laser scanning confocal microscope. Representative views were shown. Data were representative of 2 independent experiments.

On the other hand, as expected, when fluorescent ABO blood group antigen antibodies were incubated with sperm cells, blood group antigen A and B antibody can only bind to A and B type sperm samples, respectively. No immunofluorescence was detected when anti-A or anti-B antibody was incubated with sperm samples of O type and non-secretors (Fig. 3 and Supplementary Fig. 3).

### Separation of sperm cells from mixture samples by MACS and FACS

**Figure 4:**
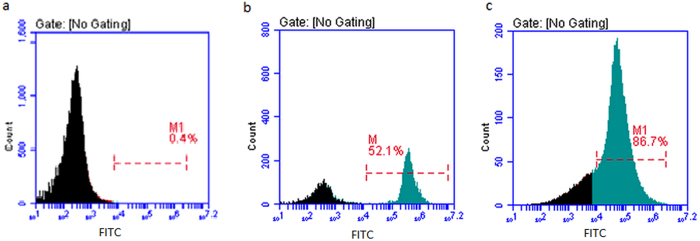
Representative data of FACS detection of MACS separated sperm cells from sperm and vaginal swab mixture sample. (**a**) Negative control (Female vaginal swab sample suspension). (**b**) Sperm cells suspension and vaginal swab sample suspension mixture before MACS separation. (**c**) Sperm cells suspension and vaginal swab sample suspension mixture after MACS separation. Numbers indicate the percentage of positive cells.

Sperm cells and female vaginal epithelial cells mixture samples were prepared and separated using FITC-conjugated anti-AKAP3 antibodies as described in Materials and Methods section. Cytofluorimetric analysis was performed to examine separation results by detecting fluorescence intensity of cells in the FITC channel and representative results were illustrated in Fig. 4. As expected, cells in the negative control sample displayed minimal fluorescence (Fig. 4a). For sperm and vaginal epithelial cells mixture samples, in spite of the presence of negative female epithelial cell populations, separation was effective and unambiguous with more than 86% cells being target sperm cells whereas only 52% positive cells were detected before MACS separation (Fig. 4b,c). Our data showed that AKAP3 is sperm specific and MACS isolation method provides enough highly purified sperm cells for DNA typing in mixtures.

**Figure 5:**
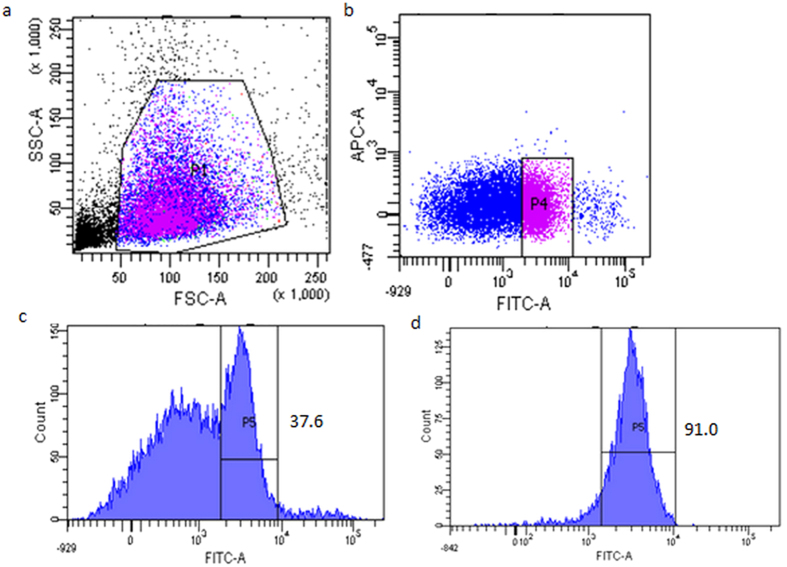
Representative cytofluorimetric analysis of the O-type sperm and A-type sperm cell mixtures using FITC-labeled anti- blood group A antigen antibody. (**a**) Gating strategy for the identification of A-type sperm cells using anti-A blood group antigen antibody. (**b**) Representative data for the detection of sperm cells in O-type and A-type sperm mixture with FITC-labeled blood group A antigen antibody. (**c**)Histogram of (**b**). (**d**) Flow cytometry analysis of A-type sperm cells after sorting. Numbers indicate the percentage of positive cells.

Representative result of the detection of sperm cells in O-type sperm and A-type sperm mixture with FITC-labeled blood group A antigen antibody is shown in Fig. 5(a–c). There is clear fluorescence-based separation between A-type sperm cells and O-type sperm cells in the mixture. We can see from Fig. 5d that about 90% of sorted cells are target sperm cells (A-type) after FACS.

### STR typing identification of sperm cells after MACS and FACS separation

STR typing was done to analyze the separation efficiency of MACS and FACS to isolate target sperm cells in mixture samples by analyzing the relative allelic contributions to the STR profiles of separated cell populations. Our data showed that only male DNA peaks were observed and a full single-source STR profile (>13 loci; >100 RFUs) could be obtained in all 30 samples isolated by MACS (Supplementary Table S1).

**Figure 6:**
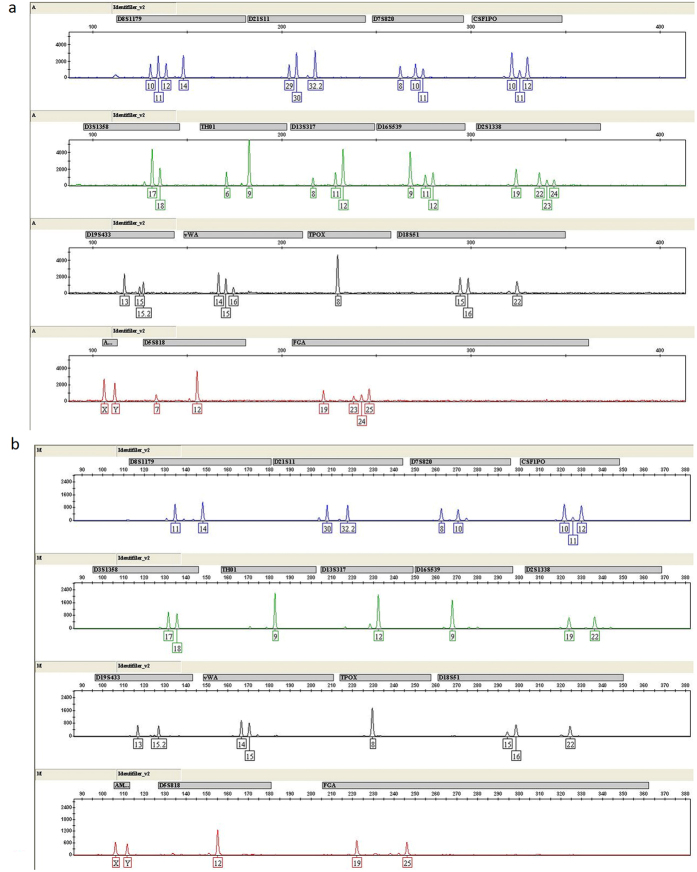
Representative CE of the STR typing with ID-plus by FACS from sperm mixtures involving two donors. (**a**) STR typing of O-type sperm and A-type sperm cell mixture. (**b**) STR typing of single-source A-type sperm cells, using blood group A antigen antibody after cell sorting.

For FACS isolated sperm cells from mixed semen samples involving plural contributors, DNA typing using the ID-plus was carried out to test the separation efficiency. As we can see from Fig. 6, a single source male DNA from sperm mixtures was successfully recovered after FACS isolation using blood group A antigen antibody. Success rate of full STR loci amplification could reach about 60% for sperm cells mixtures involving only two donors after FACS isolation (Supplementary Table S1). However, due to the need of sequential use of two antibodies, none of the sperm cells samples involving three donors gave full STR loci amplification after FACS isolation. Indeed, partial amplification (9–13 loci amplified) was obtained for all of them after FACS isolation.

### Sensitivity test using samples with varies mixed ratio

STR profiling results of varies mixed ratio showed that the sperm cells was isolated completely by MACS at as low as 1:32 mixed ratio, regardless of the large amount of background vaginal DNA present. However, selective isolation was not successful from the sample of 1:64 mixed ratio due to the effect of background vaginal cells. For FACS isolation of sperm cells mixtures, as detected by STR typing, single source B type sperm cells could be successfully separated from O type sperm cells at 1:8 mixed ratio at the most (Table 1).

### Mock sample separation by MACS and FACS

**Figure 7:**
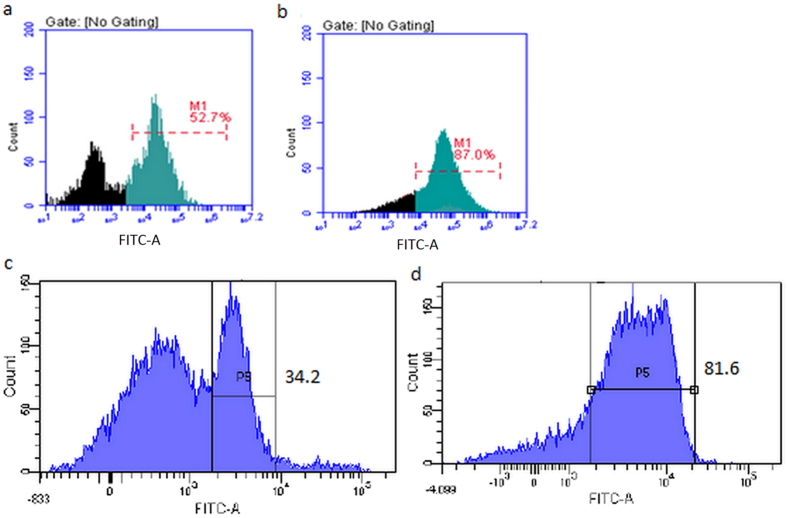
Cytofluorimetric analysis of mock sample mixture S1 involving female vaginal epithelial cells, A-type sperm cells and B-type sperm cells using FITC-labeled anti-A antigen antibody. (**a**) Sperm cells suspension and vaginal swab sample suspension mixture before MACS separation. (**b**) Sperm cells suspension and vaginal swab sample suspension mixture after MACS separation. (**c**) Detection of sperm cells in A-type and B-type sperm mixture with FITC-labeled blood group A antigen antibody. (**d**) Flow cytometry analysis of A-type sperm cells after sorting. Numbers indicate the percentage of positive cells.

Two forensic mock samples were prepared as described in Materials and Methods section. Vaginal cotton swabs containing one female vaginal epithelial cells and male sperms from two male donors were first separated by MACS using FITC-conjugated anti-AKAP3 antibodies followed FACS isolation. FITC-conjugated anti-A human group antigen antibodies were used for FACS isolation of S1 and S2, respectively. For sample S1, clear separation of A-type sperm cells from B-type sperm cells was seen after FACS with the percentage increased from 34.2% to 81.6% (Fig. 7a,b).

**Figure 8:**
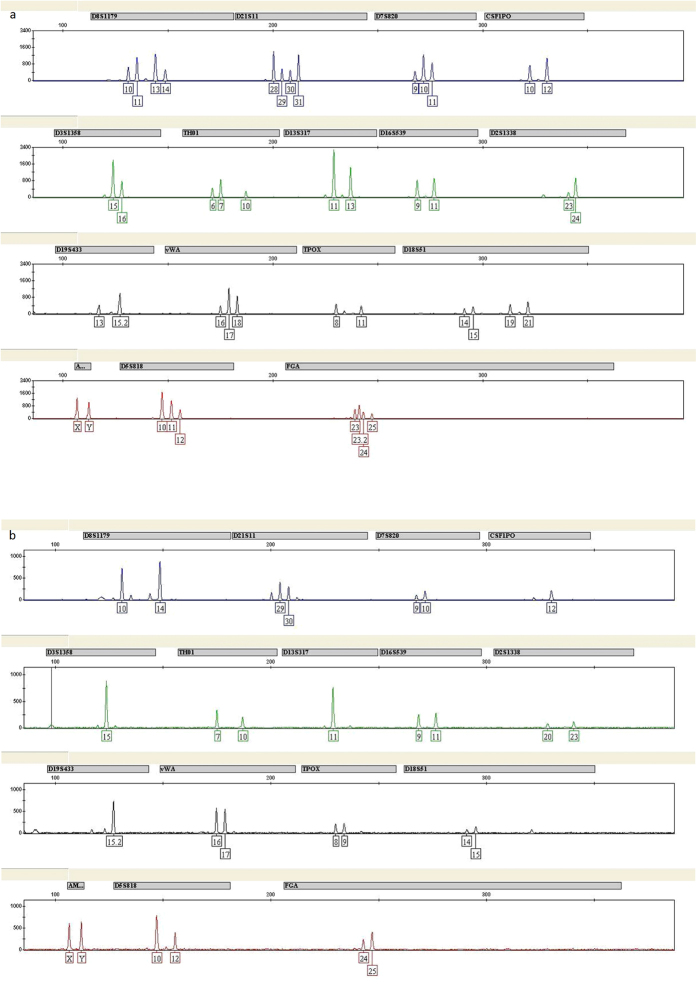
CE of the STR typing with ID-plus by FACS of mock sample mixture S1 involving female vaginal epithelial cells, A-type sperm cells and B-type sperm cells. (**a**) STR typing of A-type sperm and B-type sperm cell mixture. (**b**) STR typing of single-source A-type sperm cells, using blood group A antigen antibody after cell sorting.

We then carried out STR analysis to further confirm the separation efficiency. Although there was a minimal contribution from B-type sperm cells in the STR profiles developed from A-type sperm cells, the large difference in peak heights between the major and minor components, which allowed the complete genotype of interpretation (Fig. 8). Thus, separation of mock sample S1 by MACS and FACS produced a single source profile that was applicable for practical use. Separation results of mock sample S2 were illustrated in Supplementary Figs 4 and 5.

## Discussion

The genetic characterization of mixed biological stains remains an important area where improvement is imperative. In order to solve the problem, the present study describes two novel cell-sorting strategies for forensic analysis of male sperm DNA from mixture samples, *eg.* MACS separation of sperm cells from the vaginal swab and FACS isolation of single donor sperm cells from sperm cell mixtures involving plural contributors.

To isolate male sperm cells from mixtures involving female epithelial cells, previous studies by Anslinger *et al*.^5^ and Xue-Bo Li *et al*.^4^ showed that magnetic beads coupled to an antibody against testicular angiotensin-converting enzyme (tACE) or motile sperm domain-containing protein 3 (MOSPD3), can successfully separate sperm cells from cell mixtures. Here, we further validated the feasibility of this method for the isolation of sperm cells from sperm-female epithelial cell mixtures using magnetic beads coupled with FITC-labeled AKAP3 antibody. The location of sperm antigens determines the binding efficiency of the antibody and the sperm cells^4^. Consistent with previous studies, our study demonstrated that AKAP3 is absolutely sperm-specific (Fig. 1)^6^,^7^,^8^. However, our data showed that AKAP3 is abundantly distributed on the surface of sperm and located in the sperm head, neck, mid-piece and flagellum (Fig. 2), which is slightly different from the findings of Xu *et al*.^7^. These characteristics make AKAP3 an ideal candidate antigen for immune-magnetic bead-based isolation of sperm DNA from epithelial cells. Furthermore, the binding efficiency between the antibody and magnetic beads, as well as the optimal ratio between cell number and beads are the two key factors for successful sperm separation from vaginal swab. Flow cytometry analysis showed that AKAP3 is sperm specific and MACS isolation method provides enough highly purified sperm cells for DNA typing in mixtures (Fig. 4). However, Negative cell populations were still detected in minor quantities largely because of the non-specific binding of the vaginal epithelial cell during on-column purification. Nevertheless, STR typing analysis revealed that only male DNA peaks were observed in all mixture samples indicating that MACS separation is effective and could be applied to the isolation of forensic mixture samples (Supplementary Table S1).

Differential lysis has been the forensic standard for separating sperm DNA from vaginal or oral swabs^17^,^18^. This method relies on cell-specific differences in membrane chemical composition. Non-sperm cells are first lysed without disrupting the sperm cells and then residual exogenous DNA are washed away from the intact sperm cells^19^. However, sperm cells are prone to be lost in the first digestion and the following washing steps. Furthermore, chances for successful STR typing are limited with this approach when samples contain few spermatozoa due to over-digestion or incomplete lysis of the female’s epithelial cells. What’s more, it is obvious that differential lysis cannot be used for individual identification for samples contain mixed sperms from different offenders. On the other hand, Y-STR amplification, namely haplotype-specific extraction (HSE), has shown to be a powerful tool for the isolation of male DNA mixtures from a single contributor of male and female mixtures^20^,^21^. This strategy, however, it cannot be used to probe the autosomal STR profiles in mixtures containing more than one person of the same sex. Since paternal relatives are identical, Y-STR typing cannot be used to distinguish among brothers or even distant paternal relatives. In addition, without recombination between loci, the product rule cannot be used and thus the discrimination power of Y-STR is limited by the size of the population database used. Therefore, the application of Y-STR amplification is limited for individual identification. In our study, for unbalanced sperm/epithelial cells and sperm cells mixtures, sensitivity results revealed that target sperm cells could be detected at as low as 1:32 and 1:8 mixed ratios, respectively. Our data clearly demonstrated MACS and FACS separation is superior to conventional differential lysis and Y-STR amplification for individual identification when dealing with samples contain very few or mixed sperm cells.

In some sexual assault cases involving plural contributors of the same gender, the isolation of cells as single-source and extraction of DNA from the same cell type mixtures remains a challenge for forensic investigations. FACS is a method commonly used in biomedical research for cell sorting with fluorescent probes. In recent studies, flow cytometry has also been successfully applied to identify and separate sperm cells from vaginal epithelial cell mixtures in forensic science^9^,^22^. However, only scarce data has been published applying this approach to mixed samples from two or more donors with the same gender^11^,^12^,^23^. We, for the first time, aimed to identify single donor’s genotype from mixed sperm cells involving plural contributors by FACS. In the present study, we tried to isolate sperm mixtures from multiple donors with different ABO blood group antigens. As ABO blood group antigens are located on sperm surface^24^,^25^ and would facilitate the success of flow cytometry based sperm separation, therefore, it is imperative that we should first determine whether the genotype of semen is a secretor or non-secretor, followed by FACS separation to isolate type O, A, B, or AB sperm using ABO blood group antigen antibodies. It is known that the frequency of secretors: non-secretors is approximately 1:4, in spite of a slightly variation for different ancestry. So chances for successful application of this technique to forensic cases are quite high. Furthermore, we found that cell to antibody ratio optimization is another key factor to get the best separation result by FACS. Our FACS data showed that positive samples were successfully separated from negative samples in cell mixture and a full single-source STR profile could be obtained in most two person mixture samples after STR genotyping (Supplementary Table S1). STR analysis of mock sample mixtures separated by sequential use of MACS and FACS showed that minor peaks from the non-target contributor were indeed observed in the STR profiles of the target donor, however, there is clear major difference in the peak heights that permitted a complete genotype of the target donor (Fig. 8 and Supplementary Fig. 5).

In conclusion, our study demonstrated combined use of MACS and FACS represents an effective approach for generating single donor STR profiles from forensic mock mixture samples containing female epithelial cells and sperm cells originating from different men. However, if this method is applied to forensic cases, further optimization is needed. As ABH antigens in semen were present only at small quantities on spermatozoa as detected by flow cytometry^26^, a potential drawback of separating mixed samples by FACS using ABO antibodies is its relatively low positive percentage and non-specific interference. We found that cells and antibodies ratio as well as fluorescence-labeled antibody concentration are two key determine factors to avoid the above problems. In addition, this method relies highly on cell number (usually not less than 10^5^ cells) and intact cell structure which gives natural surface antigenic conformation, making it more suitable for the detection of fresh samples in forensic science. In addition, ABO antibody based cell-mixture separation protocol by FACS is only applicable for discriminating from a mixture of different blood types or secretor and non-secretor if ABO antibodies are used. Nevertheless, as the frequency of secretor and non-secretor is about 4:1 in the general population, the drawback mentioned above does not limit the application of this method in forensic science. Furthermore, as reported previously^11^, this technique may not be appropriate if mixture ratios are less than 1:50 (Table 1), especially when low copy number (LCN) samples are present. Sensitivity test showed that FACS separation results using ABO blood group antibodies are much better for sperm mixtures involving two contributors than those involving three. Sequential use of two antibodies and the loss of cells after multiple rounds of isolation may contribute to the above phenomenon. In this case, the joint use of other type-specific antibodies, such as HLA, and next generation DNA sequencing technologies would be considered in our future research.

## Methods

### Sample collection

This study was approved by the Ethics Committee of Fudan University, China. All the participants provided their written informed consent of the samples and the subsequent analysis. Subjects are protected by informed consent process. The investigation was conducted in accordance with humane and ethical research principles of Fudan University, China.

Semen samples were obtained with liquefaction from 80 healthy male volunteers. For these 80 male volunteers, blood samples were taken from each volunteer for ABO and DNA typing. Buccal swabs were collected from 6 healthy female volunteers. Semen-free vaginal swabs were donated from 10 healthy female volunteers. After three washings with phosphate-buffered saline buffer (PBS), all the samples were counted using a cell counter (Countess^TM^, Invitrogen, USA) to prepare cell suspensions of 10^7^ cells/mL cell densities.

To prepare mock case samples, 5 μL of semen from 2 donors with different blood type was thoroughly mixed and spotted onto semen-free vaginal swab which was donated from one healthy female volunteer and let dry at room temperature. Samples were soaked in PBS to prepare cell suspension. Mock sample one (S1) contains sperm cells from donors with A and B blood type. Mock sample two (S2) contains sperm cells from donors with O and AB blood type.

### ABO and STR genotyping

For sperm cells samples, ABO and STR typing were first done according to the method described previously^16^. DNA was directly extracted from each semen sample using DNA IQ^TM^ (Promega, USA) and eluted in 50 μL Elution buffer following manufacturer’s instructions. DNA extracts were quantified using the Quantifiler system (Applied Biosystems, USA). Real-time PCR were carried out with an ABI 7500 real-time PCR machine according to the manufacturer’s instructions. Extracted DNA were adjusted to the final concentration to 1 ng/μL. Two microliters DNA was co-amplified using ABO and STR primer sets with Golden*e*ye^TM^ DNA ID System 16 BT kit (Peoplespot, Beijing, China), according to the manufacturer’s protocol. Amplification was performed in a reaction volume of 25 μL. PCR products were analyzed by capillary electrophoresis (CE) on a laser-induced fluorescence ABI Prism 3130xl Genetic Analyzer (Applied Biosystems). A blank composed of nuclease-free water and 9947A was included as a control. Raw data were analyzed using GeneMapper Software Version 3.2 (Applied Biosystems), where a peak detection threshold of 100 relative fluorescence units (RFUs) was used^16^.

### Secretors or non-secretors identification of sperm cells

DNA was extracted and quantified as described in the above section. The FUT2 gene (Genebank number: NM000511.5) was amplified on a GeneAmp9700 PCR System (Applied Biosystems) using the following primers: FUT2-F: ACCTGAACGACTGGATGGAG, FUT2-R: CACCATCTACCTGGCCAATT. The resulting 550 bp PCR products were purified before sequencing.

### Western blot

Sperm cell suspensions at a density of 10^5^ cells/mL and the buccal/vaginal epithelial cell suspensions of 10^4^ cells/mL were used for Western blot analysis. Total protein extracts were collected by using cell lysis buffer for western or IP (Beyotime, #P0013, China). The total protein concentration of each sample was measured using an enhanced BCA protein Assay Kit (Beyotime, #P0009, China). Equal amounts of total proteins (60 μg) from each sample were resolved via 10% SDS-PAGE gel and transferred to a nitrocellulose filter membrane 200 mA for 1 hr at ice-water mixture. The blot was blocked with 5% non-fat dry milk in PBST (500 mL 1 × PBS + 1 mL Tween20) for 1 hr at room temperature and then incubated with 1:500 diluted anti-AKAP3 monoclonal antibodies (Abcam, #170856, UK) and 1:2000 diluted anti-GAPDH monoclonal antibodies (Cell Signaling Technology, #5174, USA) separately overnight at 4 °C. On the next day the blots were washed three times with PBST for 10 mins each time followed by incubation with secondary HRP-linked antibody (Cell Signaling Technology, #7074S, 1:2000, USA) for 1 hr at room temperature. Bands were scanned using a densitometer (Bio-Rad, USA).

### Immunofluorescence

For sperm/female epithelial cell mixtures, 0.1 mL of sperm cell suspension (10^5^ cells/mL) or buccal/vaginal epithelial cell suspension (10^4^ cells/mL) was added to a slide, dried in an incubator at 37 °C and fixed with 4% paraformaldehyde for 15 mins at room temperature. After three washings, the samples were blocked with blocking buffer (50 mL PBS, 1.5 mL FBS, 0.5 mL Goat-serum, 0.05 mL Triton X-100) for 2 hrs at room temperature. Subcellular localization of AKAP3 was determined by co-staining sperm cells with antibodies against a cell surface antigen marker ATP1A1. Sperm cells were detected with 1:100 diluted anti-AKAP3 monoclonal antibodies (Abcam, #170856, UK) and anti-ATP1A1 polyclonal antibodies (Proteintec, #55187-1-AP, USA). Rabbit IgG monoclonal isotype control antibody (Abcam, #ab172730, UK) was stained in parallel as negative controls. Primary antibody binding was detected by incubating the slides with 1:500 diluted Alexa Fluor 488 conjugated anti-rat IgG (H+L) secondary antibodies (Cell Signaling Technology, #4416, USA) and Alexa Fluor 594 conjugated goat anti-rabbit secondary antibodies (Proteintec, SA00006-4, USA) for 1 hr at temperature. Between the first and second incubation, the slides were washed three times with PBS for 5 mins each time. Nuclei were counterstained with 4′, 6-diamidino-2-phenylindole (DAPI) for 10 mins, and then the slides were observed under a laser scanning confocal microscope (Zeiss, LSM710, Germany).

For mixed sperm, immunofluorescence was carried out as described above using 1:100 diluted blood group A antigen monoclonal antibody (Santa Cruz, sc-69951, USA) and blood group B antigen monoclonal antibody (Santa Cruz, sc-69952, USA). Normal mouse IgM-FITC (sc-2859) were used as negative control antibody and stained in parallel.

### Isolation of sperm cells from sperm cells and female vaginal epithelial cells mixtures by magnetic separation

Sperm cells and female vaginal epithelial cells mixture samples used for MACS separation were prepared as follows. Sperm cell suspensions and vaginal epithelial cell suspensions were first adjusted with PBS to 10^6^ cells/mL, respectively. Sperm cells and female vaginal epithelial cells mixture were prepared by adding 1 mL sperm cell suspension to 1 ml of vaginal epithelial cell suspension. Three sperm cell suspensions and 10 vaginal epithelial cell suspensions were mixed randomly so that a total of 30 mixed sperm and female cell samples were obtained.

Five microliters FITC-conjugated AKAP3 polyclonal antibody (PR-8197, 1 mg/mL, HopeBiot, China) or FITC labeled isotype control antibody was incubated with 100 μL Anti-FITC MicroBeads (Miltenyi Biotec, GmbH, Germany) for 2 hrs at 20 °C, followed by 3 washings with 1.5 mL PBS. The resulting microbeads were referred to AKAP3-coupled immune-magnetic beads in the rest of the article.

Subsequently, 40 μL AKAP3-coupled immune-magnetic beads was added to 300 μL cell mixture suspension (5 × 10^5^ cells/mL) in PBS containing 10% FBS, 2 mM EDTA and incubated at 37 °C for 2 hrs on a Thermomixer comfort (Eppendorf, Germany) at 800 rpm/min. The unbound primary antibody was removed by washing with 1.5 mL PBS and centrifuged at 300 g for 10 mins. The washing step was repeated twice. Magnetic separation was performed with MS Columns and MACS Separator (Miltenyi Biotec, GmbH, Germany), according to the manufacturer’s instructions. After separation, the FITC-labeled cells were detected by flow cytometry (AccuriC6, BD Biosciences, USA).

### FACS sorting sperm cells mixtures involving two or three donors by ABO blood type antigen antibody

Donors to prepare sperm cell mixtures are selected according their blood group type and secretor status analysis. Sperm cell mixtures involving two or three donors were prepared as listed in Table 2. Sperm cell suspensions were first adjusted with PBS to 10^6^ cells/mL. 1 mL sperm cell suspension from each of the two or three donors were mixed together to reach a 1:1 ratio. Blood group antibodies used to separate sperm cell mixture involving plural contributors are listed in Table 1. Sperm cell mixture suspensions were first filtered through 40 μM Cell Strainer (BD Falcon, #352340, USA) followed by staining with fluorescent antibodies. Briefly, 6.8 μL FITC-labeled blood group A or 9 μL FITC-labeled blood group B antigen antibodies was added to 300 μL 5 × 10^5^ cells/mL sperm cell suspension suspended in PBS containing 10% FBS, 2 mM EDTA and incubated at 37 °C for 1 hr. FITC-labeled isotype control antibodies were used as control when performing FACS separation. The unbound primary antibody was removed by washing with 1.5 mL PBS and centrifuged at 400 g for 10 mins. Labelled cells were then sorted with a FACSAria^TM^ II sorter (70 μm nozzle; BD Biosciences, USA). Positive sperm cells were collected directly into Lysis Buffer (Promega, USA).

### DNA extraction and STR profiling

DNA extraction and quantification was done as described above. Two microliters DNA (or up to 10 μL, if the 2 μL result was weak) were amplified with the AmpF*l*STR Identifiler Plus PCR amplification kit (ID-plus, Applied Biosystems), on a GeneAmp PCR System 9700 (Applied Biosystems) in a total reaction volume of 25 μL according to the manufacturer’s protocol. PCR products were analyzed by CE as previously described^27^. Based on STR typing using blood samples, the number of samples that had a full single-source STR profile was calculated. The number of correctly amplified gene loci was also recorded. A threshold value of 100 relative fluorescence units (RFU) was used to confirm successful typing. For sperm/vaginal mixture and sperm mixture specimens, detection was deemed full amplification when successful typing was achieved more than 13 loci out of 16 amplified loci, whereas detection was considered partial amplification when the number of successfully amplified loci is 9–13, low partial when the number of successfully amplified loci is 3–8 and none when the number of successfully amplified loci is less than 3.

### Sensitivity test using samples with varies mixed ratio

Mixture experiments were carried out to simulate conditions in which a small amount of target cells must be discriminated from a high background of other types of cells. Sperm cells and vaginal epithelial cells mixture were mixed at variable ratios. Experimentally diluted sperm cells and vaginal epithelial cells were mixed in the ratios of 1:1, 1:4, 1:16, 1:32 and 1:64 in the test tube. Sperm cells and vaginal epithelial cells mixtures were later stained with anti-AKAP3 antibody and separated using MACS. For sperm cell mixtures, sperm cells of B and O type were mixed at variable ratios. Experimentally diluted B and O type sperm cells were mixed in the ratios of 1:1, 1:2, 1:4, 1:8 and 1:16 in the test tube. Sperm cell mixtures were later stained with anti-B blood group antigen antibodies and separated using FACS.

## Additional Information

**How to cite this article**: Xu, Y. *et al*. Fluorescence- and magnetic-activated cell sorting strategies to separate spermatozoa involving plural contributors from biological mixtures for human identification. *Sci. Rep.* **6**, 36515; doi: 10.1038/srep36515 (2016).

**Publisher’s note**: Springer Nature remains neutral with regard to jurisdictional claims in published maps and institutional affiliations.

## Supplementary Material

Supplementary Information

## Figures and Tables

**Table 1:** Number of STR loci successfully amplified from sperm and epithelial cell mixtures (MACS) and sperm cell (B and O types) mixtures (FACS) mixed at different ratios (n = 8).

Mixture ratios	Number of STR loci successfully amplified (RFU ≥ 100)			
	14–16 (full)	9–13 (partial)	6–8 (low partial)	<6 (none)
1:1(s:v) by MACS	8/8	—	—	—
1:4(s:v) by MACS	7/8	1/8	—	—
1:16(s:v) by MACS	5/8	3/8	—	—
1:32(s:v) by MACS	3/8	4/8	1/8	—
1:64(s:v) by MACS	—	6/8	1/8	1/8
1:1(B:O) by FACS	6/8	2/8	—	—
1:2(B:O) by FACS	5/8	2/8	1/8	—
1:4(B:O) by FACS	2/8	5/8	1/8	—
1:8(B:O) by FACS	1/8	4/8	1/8	2/8
1:16(B:O) by FACS	—	2/8	2/8	4/8

s:v (sperm/vaginal) B:O (sperm of B type/sperm of O type).

**Table 2:** Blood group antibodies used to separate sperm cell mixture involving plural contributors.

Antibody	Sperm cell mixture from two different blood group individuals					
	A/B	A/O	A/AB	B/O	B/AB	O/AB
anti-A	√	√	—	—	√	—
anti-B	—	—	√	√	—	√
**Antibody**	**Sperm cell mixture from three different blood group individuals**					
	**A, O and AB**		**B, O and AB**	**A, B and AB**	**A, O and B**	
anti-A then anti-B	—		√	√	—	
anti-B then anti-A	√		—	—	√	
