# Supplementary material for: Fluorescence- and magnetic-activated cell sorting strategies to separate spermatozoa involving plural contributors from biological mixtures for human identification
Source: Sci Rep. 2016 Nov 18;6:36515. doi: 10.1038/srep36515 (PMC5114643; doi:10.1038/srep36515)
Supplement: Supplementary Information [file srep36515-s1.pdf]

Supplementary Information for:

Title: Fluorescence- and magnetic-activated cell sorting strategies to separate spermatozoa involving plural contributors from biological mixtures for human identification

Authors: Yan Xu[b,c], Jianhui Xie[c], Ronghua Chen[b], Yu Cao[b], Yuan Ping[b], Qingwen Xu[b], Wei Hu[b], Dan Wu[b], Lihua Gu[b], Huaigu Zhou[b], Xin Chen[b], ZiQin Zhao[c], Jiang Zhong[a], Rui Li[a*]

Affiliations:

[a]School of Life Sciences, Fudan University, 2005 Songhu Road, Shanghai 200438, China.

[b]Shanghai Key Laboratory of Crime Science Evidence, Key Laboratory of Forensic Evidence and Science Technology, Ministry of Public Security, Institute of Forensic Science, Shanghai Public Security Bureau, Shanghai 200083,China.

[c] Department of Forensic Medicine, Shanghai Medical College, Fudan University, 138 Yixueyuan Road, Shanghai 200032, China.

[*]**Corresponding author:** Tel.: +86 21 51630660; Fax: +86 21 51630661. E-mail address: rui_li@fudan.edu.cn (R. Li);

337tggcagaact accacctgaa cgactggatg gaggaggaat accgccacat cccgggggag
397tacgtccgct tcaccggcta cccctgctcc tggaccttct accaccacct ccgccaggag
457atcctccagg agttcaccct gcacgaccac gtgcgggagg aggcccagaa gttcctgcgg
517ggcctgcagg tgaacgggag ccggccgggc acctttgtag ggtccatgt tcgccgaggg
577gactatgtcc atgtcatgcc aaaagtgtgg aagggggtgg tggccgaccg gcgataccta
637cagcaggccc tggactggtt ccgagctcgc tacagctccc tcatcttcgt ggtcaccagt
697aatggcatgg cctggtgtcg ggagaacatt gacacctccc acggtgatgt ggtgtttgct
757ggcgatggca ttgagggctc acctgccaaa gattttgctc tactcacaca gtgtaaccac
817accatcatga ccattgggac gttcgggatc tggggccgcat acctcacggg cggagacacc
877 atctacctgg ccaattacac cctccccgac tcccctttcc tcaaaatctt

Figure S**1. Partial sequence of FUT2 gene that contain 5 mutation sites.**

Green highlights represent primer sequence. Pink highlights represent mutations that lead to inactivation of Se enzyme.

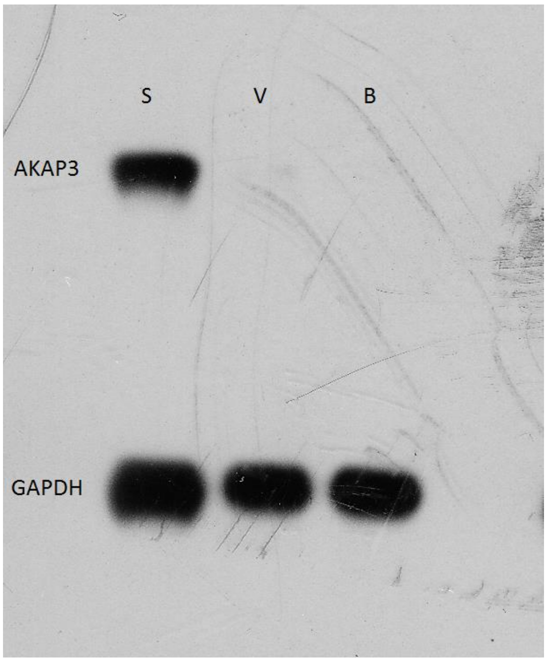

Figure S2. Full-length gels and blots of Figure 1.

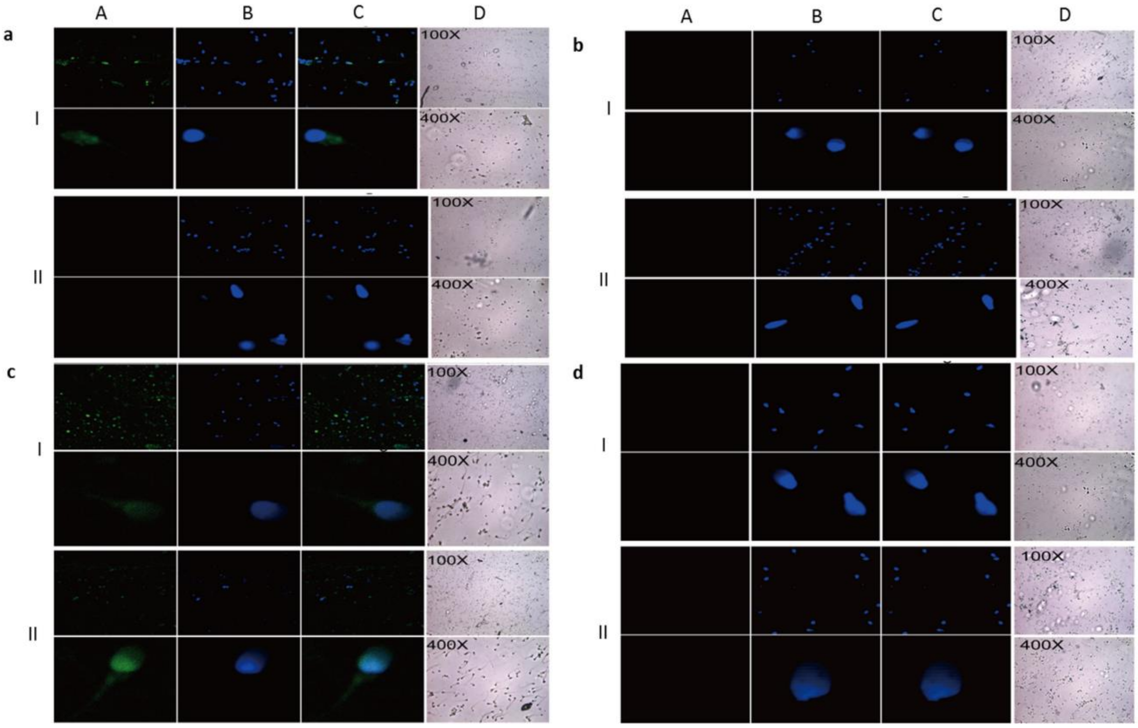

Figure S3. Immunofluorescent localization of blood group antigens in A-type (a), O- type (b), AB-type (c) and non-secretors sperm cells (d).

I. **(A)** Blood group A-stained sperm cells. **(B)** DAPI-stained sperm nuclei. **(C)** Merged image (A, B). **(D)** Sperm cells under a light microscope (100× and 400×).

II. **(A)** Blood group B-stained sperm cells. **(B)** DAPI-stained sperm nuclei. **(C)** Merged image (A, B). **(D)** Sperm cells under a light microscope (100× and 400×).

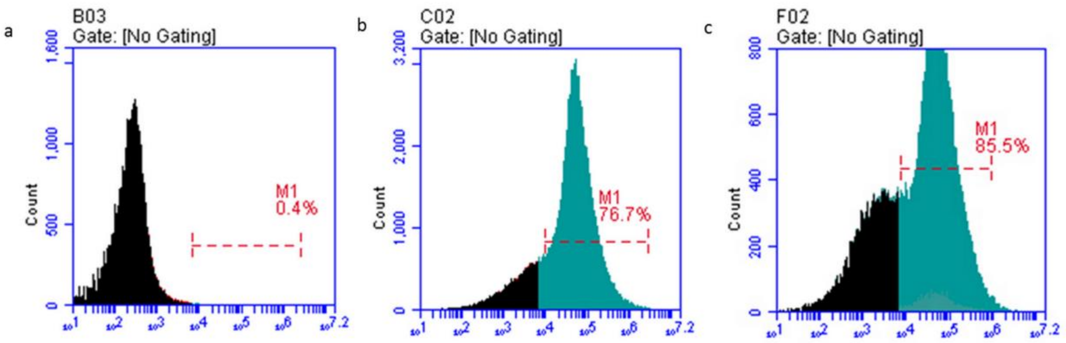

Figure S4. Cytofluorimetric analysis of mock sample mixture S2 involving female vaginal epithelial cells, AB-type sperm cells and O-type sperm cells using FITC-labeled anti- blood group A antigen antibody.

(a) Negative control (Female vaginal swab sample suspension).

(b) Detection of sperm cells in AB-type and O-type sperm mixture with FITC-labeled blood group A antigen antibody.

(c) Flow cytometry analysis of A-type sperm cells after sorting.

Numbers indicate the percentage of positive cells.

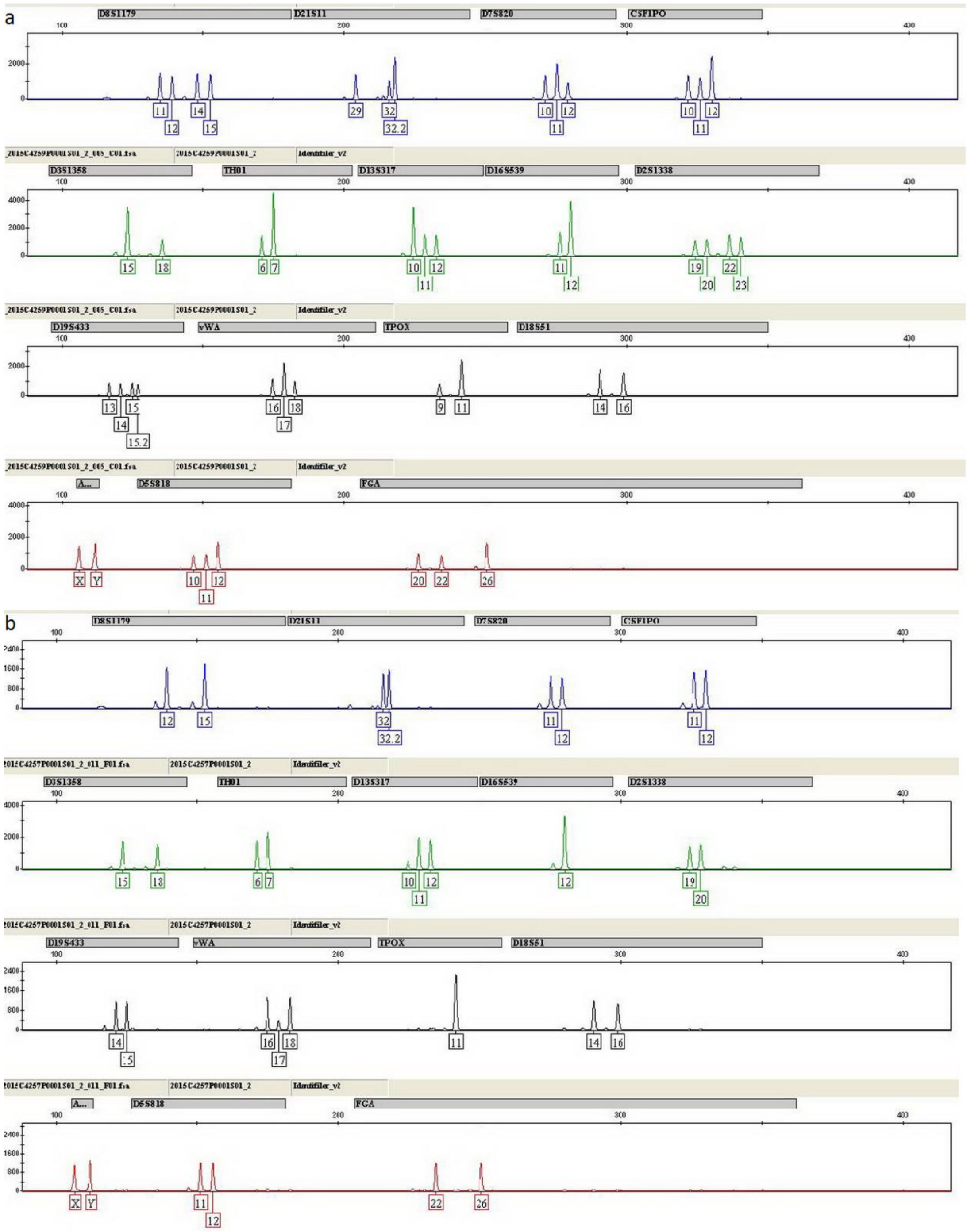

Figure S5. CE of the STR typing with ID-plus by FACS of mock sample mixture S2 involving female vaginal epithelial cells , AB-type sperm cells and O-type sperm cells.

(a) STR typing of AB-type sperm and O-type sperm cell mixture.

(b) STR typing of single-source AB-type sperm cells, using blood group A antigen antibody after cell sorting.

Table S1. Success rate of STR loci amplification after cell mixtures incubated with different antibodies (mixtures ratio at 1:1)

Antibody (cell mixture type)	Success rate of STR loci amplification (RFU⩾100)			
	14-16 (full)	9-13 (partial)	6-8 (low partial)	<6 (none)
AKAP3 (s:v) (n=30)	30	0	0	0
ABO (A:B) (n=6)	4	2	0	0
ABO (A:O) (n=6)	4	2	0	0
ABO (A:AB) (n=6)	4	2	0	0
ABO (B:O) (n=6)	4	2	0	0
ABO (B:AB) (n=6)	3	3	0	0
ABO (O:AB) (n=6)	4	2	0	0
ABO (A:O:AB) (n=6)	0	6	0	0
ABO (B:O:AB) (n=6)	0	6	0	0
ABO (A:B:AB) (n=6)	0	6	0	0
ABO (A:O:B) (n=6)	0	6	0	0

s:v (sperm and vaginal epithelial cell); A:B (sperm of A type and sperm of B type)
A:O:B (sperm of A type, sperm of O type and sperm of B type)
